# Long-Term Enhancement of Botulinum Toxin Injections for Post-Stroke Spasticity by Use of Stretching Exercises—A Randomized Controlled Trial

**DOI:** 10.3390/toxins16060267

**Published:** 2024-06-11

**Authors:** In-Su Hwang, Jin-Whan Ryu, Sol Jin, Soo-A Kim, Min-Su Kim

**Affiliations:** 1 Department of Rehabilitation Medicine, Soonchunhyang University Cheonan Hospital, Cheonan 31151, Republic of Korea; 135829@schmc.ac.kr (I.-S.H.); 129751@schmc.ac.kr (J.-W.R.); 138411@schmc.ac.kr (S.J.); sooapmr@schmc.ac.kr (S.-A.K.); 2 Department of Regenerative Medicine, College of Medicine, Soonchunhyang University, Cheonan 31151, Republic of Korea

**Keywords:** dystonia, pain, physical therapy, rehabilitation, spasticity, stretching, stroke

## Abstract

Botulinum toxin A (BONT/A) injections play a central role in the treatment of upper limb spasticity in stroke patients. We proposed structured stretching exercises to enhance the effect of post-stroke spasticity relief of the upper limbs following BONT/A injections. A total of 43 patients who had a stroke with grade 2 spasticity or higher on the Modified Ashworth Scale (MAS) in their upper-limb muscles were randomly assigned to the intervention (*n* = 21) or control group (*n* = 22). The former received structured stretching exercises after their BONT/A injections for 20 min, 5 days per week, for 6 months at a hospital, while the others conducted self-stretching exercises at home. The outcome measures were assessed before the intervention (T0) and after three (T1) and six months (T2). Significantly greater improvements in the MAS scores of the elbows, wrists, and fingers were found in the intervention group’s patients at T1 and T2. The behavioral outcome measures, including shoulder pain, activities of daily living, and quality of life, and our electrophysiological studies also showed a significantly higher enhancement in this patient group. In conclusion, the structured stretching exercises plus BONT/A injections for six months showed a superior effect in relieving post-stroke upper-limb spasticity compared to self-stretching exercises.

## 1. Introduction

Spasticity is an upper-motor neuron syndrome defined as the hyperexcitability of muscles resulting in an increase in stretch reflexes, characterized by excessive tendinous reflexes, significant resistance to passive movement, and hypertonia [1]. The prevalence of spasticity after a stroke has been reported to range from 4 to 42% within one year after onset [2,3,4], and its symptoms are known to generally become prominent in the first three months after stroke onset [5,6]. Because spasticity incidence is associated with stroke severity and muscle weakness degree [7,8], it has been reported in 25–50% of patients who have experienced a stroke and have severe sensorimotor impairments [9]. Post-stroke spasticity significantly reduces independence in activities of daily living (ADL) because it reduces joints’ range of motion and prevents the patient from voluntarily moving their arms and legs [8]. In particular, patients who have experienced a stroke and have upper-limb spasticity complain of severe shoulder pain on the affected side as well as in the other fingers and wrist joints [10]. As a result, post-stroke spasticity has a substantial impact on a patient’s chances of success in rehabilitation and their functional outcomes after a stroke [5,11].

Botulinum toxin type A (BONT/A) is an injectable drug, with a peripheral mechanism of action, that can effectively treat upper-limb spasticity among muscle agonists [12]. Several randomized, double-blind, placebo-controlled trials on the effects of BONT/A in treating spasticity after a stroke have reported a significant reduction in muscle tone in the wrist, elbow, fingers, and thumb [5,13]. Although BONT/A injections have been established as the gold-standard treatment for post-stroke spasticity, the duration (usually 3–6 months) and degree of spasticity relief vary depending on the patient [12,14,15]. Therefore, several adjuvant treatments have been proposed to enhance BONT/A injections’ post-stroke spasticity relief effect. Physical therapy, casting and taping, functional electrical stimulation, and robotic rehabilitation have all been applied to patients with lower-limb post-stroke spasticity as adjunctive treatments to BONT/A injections, and motor function and walking speed improvements have been shown [16,17,18]. Several recent studies have shown that photobiomodulation, the application of which is gradually expanding [19,20], helps reduce upper-limb spasticity after a stroke [21,22].

Still, few studies have investigated the efficacy of BONT/A plus stretching in treating upper limb spasticity. Bumbea et al. [23] divided patients with ischemic and hemorrhagic stroke into two groups—those who received botulinum toxin injections and those who received only physical therapy, such as electrical stimulation and radial shockwave therapy—examining the therapeutic effect on relieving spasticity between the two groups. Both groups were instructed to perform self-stretching exercises at home [23] and, after nine months of follow-up, the patients who received botulinum toxin injections and stretching at home showed better spasticity relief, confirming that physical therapy plus stretching exercises without botulinum toxin injections was ineffective in reducing spasticity [23]. However, rehabilitation therapy in combination with BONT/A for patients with chronic strokes has also been reported to have a similar effect to BONT/A injection alone in terms of relieving spasticity [24]. A meta-analysis study that analyzed the combined treatment effects of BONT/A and physical therapy suggested that it would be better than BONT/A alone but concluded that high-quality studies are still lacking [25].

One of the reasons why physical therapy combined with BONT/A injections for upper-limb spasticity has conflicting results may be that the method of physical therapy varies depending on the researcher. In particular, stretching is an essential spasticity rehabilitation technique [25], but previous studies have not explicitly described in detail how it was implemented. Many different ways to stretch exist, and, if they are not performed correctly or for the correct purposes, they will not be effective in relaxing the muscles [26]. Therefore, we proposed and investigated the use of structured stretching exercises that could be performed after BONT/A injections to enhance the long-term post-stroke upper-limb spasticity relief effect of the latter.

## 2. Results

### 2.1. Demographic and Clinical Characteristics of the Participants

**Figure 1:**
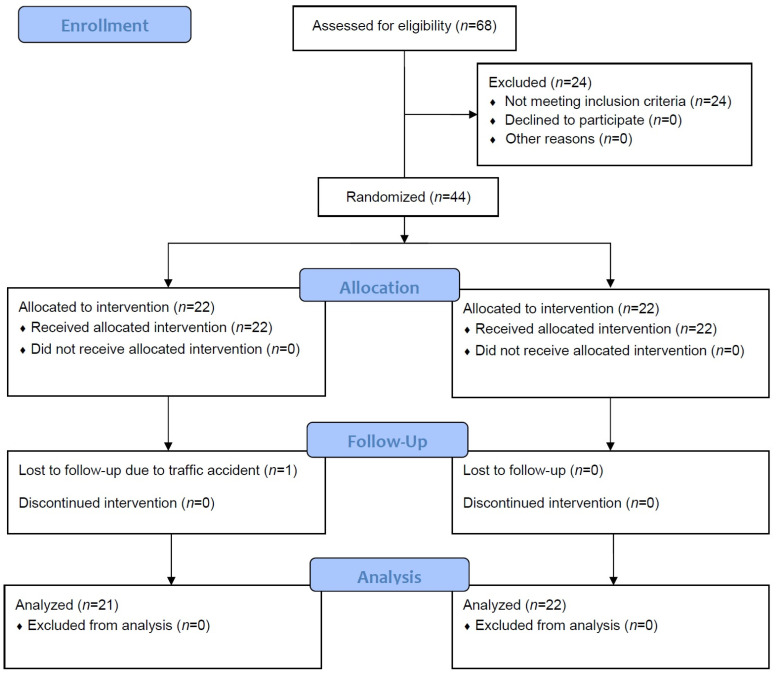
CONSORT flow diagram of recruitment to, allocation within, and participation in this study.

Sixty-eight patients were assessed for eligibility (Figure 1). Twenty-four of them were excluded as they did not meet the inclusion criteria, and one patient was lost to follow-up due to a motor vehicle accident during the intervention period. Therefore, 43 patients were included in the final analysis (*n* = 21 in the intervention group and *n* = 22 in the control group).

The checklist suggested that the patients in the control group complied well with the stretching exercises in terms of the prescribed duration and method daily for three months after the BONT/A injections. The mean age of the participants was 66.3 ± 8.2 years, and the mean period from stroke onset to treatment was 68.1 ± 8.4 days (Table 1). The two study groups had no significant differences in their demographic and clinical characteristics.

### 2.2. Effect on Primary Outcome Measures

**Figure 2:**
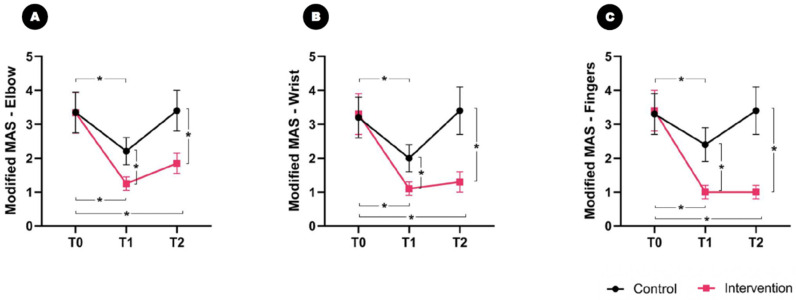
Comparison of the Modified Ashworth Scale (MAS) scores between the intervention group and the control group over time. Significant time and group interaction effects are found in the modified MAS scores of the elbows, wrists, and fingers. In the between-group comparison, the spasticity relief effect of BONT/A continues until the 6-month time point (T2) in the intervention group of patients. In contrast, the anti-spastic effect of BONT/A is almost lost at 6 months in the control patients. (**A**) Elbow; (**B**) wrist; and (**C**) fingers. * *p* < 0.05.

No significant differences between the groups were observed in the baseline modified upper-limb joint MAS scores. A significant time and group interaction effect on the modified MAS scores of the elbows, wrists, and fingers was found (F_2,33_ = 14.140, *p* = 0.032; F_2,33_ = 15.816, *p* = 0.024; and F_2,33_ = 24.930, *p* = 0.016, respectively) (Figure 2). The F-value in the repeated measures ANOVA represented the ratio of the variance between the groups to the variance within the groups: the greater the difference in results between the groups, the higher the F-value.

According to the post hoc analysis, the modified MAS score of the elbow significantly improved from 3.3 ± 0.6 at T0 to 1.2 ± 0.2 at T1 and 1.8 ± 0.3 at T2 in the intervention group (Figure 2A). In the control group, the modified MAS score of the elbow decreased from 3.3 ± 0.7 at T0 to 2.2 ± 0.4 at T1; however, it reverted to 3.4 ± 0.6 at T2. The between-group analysis showed a significant difference with respect to the change in the modified MAS scores of the elbow at T1 (intervention group: ΔT1 = 2.2; control group: ΔT1 = 1.1; *p* = 0.004) and T2 (intervention group: ΔT2 = 1.5; control group: ΔT2 = −0.1; *p* < 0.001).

Similar to the modified MAS scores of the elbow, those of the wrist significantly improved from 3.3 ± 0.6 at T0 to 1.1 ± 0.2 at T1 and 1.3 ± 0.3 at T2 in the intervention group (Figure 2B). In the control group, they changed from 3.2 ± 0.6 at T0 to 2.0 ± 0.4 at T1 and 3.4 ± 0.7 at T2. A significant difference was found between the intervention and the control group for the change in the modified MAS scores of the wrist at T1 (intervention group: ΔT1 = 2.2; control group: ΔT1 = 1.2; *p* = 0.008) and T2 (intervention group: ΔT2 = 1.9; control group: ΔT2 = −0.2; *p* < 0.001).

Additionally, the modified MAS scores of the fingers significantly improved from 3.4 ± 0.6 at T0 to 1.0 ± 0.2 at T1 and 1.0 ± 0.2 at T2 in the intervention group (Figure 2C). In the control group, they changed from 3.3 ± 0.6 at T0 to 2.4 ± 0.5 at T1 and 3.4 ± 0.7 at T2. The between-group analysis showed a significant difference between the groups with respect to the change in the modified MAS scores of the fingers at T1 (intervention group: ΔT1 = 2.4; control group: ΔT1 = 0.9; *p* = 0.004) and T2 (intervention group: ΔT2 = 2.4; control group: ΔT2 = −0.1; *p* < 0.001).

### 2.3. Effect on Secondary Outcome Measures

**Figure 3:**
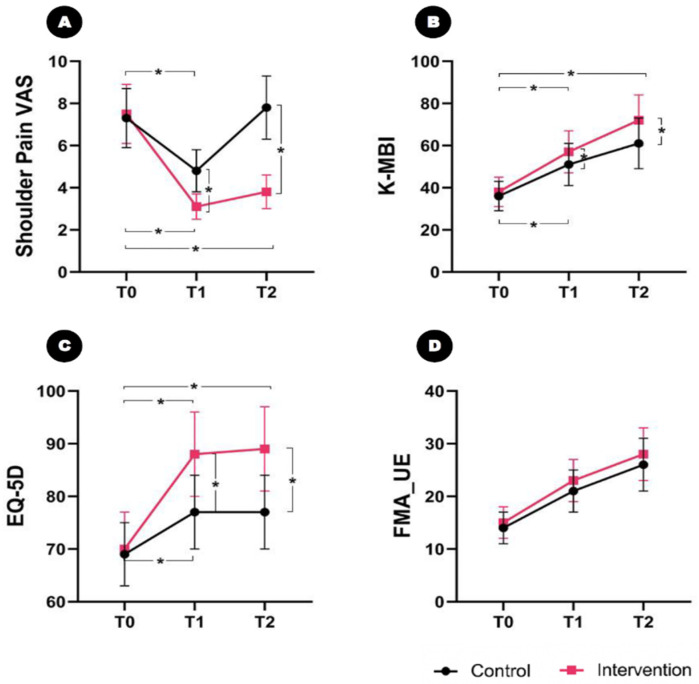
Comparison of the secondary outcome indicators between the intervention and control groups over time. Significant time and group interaction effects are found in the VAS scores of pain in the affected shoulder, K-MBI, and EQ-5D over time. The patients in the intervention group show greater improvements in their VAS, K-MBI, and EQ-5D scores at T1 and T2. However, no time and group interaction effect are found in FMA_UE. (**A**) VAS of shoulder pain; (**B**) K-MBI; (**C**) EQ-5D; and (**D**) FMA_UE. * *p* < 0.05. VAS, Visual Analogue Scale; K-MBI, Korean version of the Modified Barthel Index; EQ-5D, EuroQol-5 Dimension (EQ-5D); and FMA_UE, Fugl-Meyer Assessment of Upper Extremities.

No significant difference in the baseline VAS scores of pain in the affected shoulder, K-MBI, FMA_UE, and EQ-5D was found between the groups. After the intervention, a significant time–group interaction effect was observed in the VAS scores over time (F_2,33_ = 9.438, *p* < 0.001) (Figure 3A).

The post hoc analysis revealed that the VAS scores of pain in the affected shoulder decreased from 7.5 ± 1.4 at T0 to 3.1 ± 0.6 at T1 and 3.8 ± 0.8 at T2 in the intervention group. In the control group, the VAS score changed from 7.3 ± 1.4 at T0 to 4.8 ± 1.0 and 7.8 ± 1.5 at T2. The between-group analysis showed a significant difference between the groups with respect to the change in the VAS score of the affected shoulder at T1 (intervention group: ΔT1 = 4.4; control group: ΔT1 = 2.5; *p* = 0.004) and T2 (intervention group: ΔT2 = 3.7; control group: ΔT2 = −0.5; *p* < 0.001).

A significant time and group interaction effect was found in the K-MBI (F_2,33_ = 8.120, *p* = 0.016) (Figure 3B). The K-MBI changed from 38 ± 7 at T0 to 57 ± 10 at T1 and 72 ± 12 at T2 in the intervention group. It also improved from 36 ± 7 at T0 to 51 ± 10 at T1 and 61 ± 11 at T2 in the control group. A significant difference between the groups was found according to the post hoc study for the change in the K-MBI at T1 (intervention group: ΔT1 = 19; control group: ΔT1 = 15; *p* = 0.036) and T2 (intervention group: ΔT2 = 34; control group: ΔT2 = 25; *p* = 0.032).

In addition, a significant time–group interaction effect in the EQ-5D was observed (F_2,33_ = 12.324, *p* = 0.002) (Figure 3C): it changed from 70 ± 7 at T0 to 88 ± 8 at T1 and 89 ± 8 at T2 in the intervention group and improved from 69 ± 6 at T0 to 77 ± 7 at T1 and 77 ± 8 at T2 in the control group. A significant difference was found between the groups according to the post hoc study with respect to the change in the K-MBI at T1 (intervention group: ΔT1 = 18; control group: ΔT1 = 8; *p* = 0.008) and T2 (intervention group: ΔT2 = 19; control group: ΔT2 = 8; *p* = 0.008).

However, no significant time and group interaction effect was found in the FMA_UE (F_2,33_ = 6.295, *p* = 0.103) (Figure 3D): it significantly improved from 15.0 ± 3.2 at T0 to 23.3 ± 4.4 at T1 and 28.5 ± 5.1 at T2 in the intervention group and from 14.2 ± 3.8 at T0 to 21.5 ± 4.6 at T1 and 26.4 ± 5.5 at T2 in the control group. No significant difference was found between the groups according to the post hoc study with respect to the change in the FMA_UE at T1 (intervention group: ΔT1 = 8.3; control group: ΔT1 = 7.3; *p* = 0.235) and T2 (intervention group: ΔT2 =13.5; control group: ΔT2 = 12.2; *p* = 0.214).

### 2.4. Quantitative Analysis of Spasticity Using Electromyography

**Figure 4:**
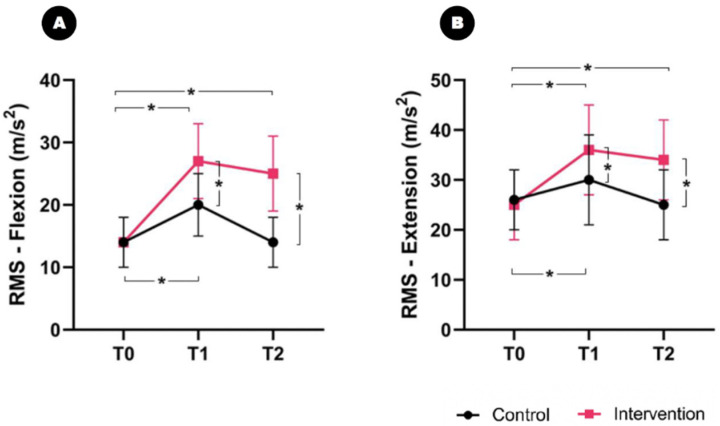
Comparison of the root mean square (RMS) using electromyography between the groups. A significant time and group interaction effect is found for RMS flexion and extension over time. The patients in the intervention group show greater enhancement in RMS flexion and extension than those in the control group. A higher RMS means that spasticity relief has been achieved. (**A**) RMS flexion; and (**B**) RMS extension. * *p* < 0.05.

No significant differences were found in the baseline RMS between the groups. However, a significant time–group interaction effect was observed in RMS flexion and extension over time after the intervention (F_2,33_ = 7.330, *p* = 0.032 and F_2,33_ = 6.251, *p* = 0.032): the former increased from 14.1 ± 4.1 at T0 to 27.4 ± 6.6 at T1 and 25.6 ± 6.9 at T2 in the intervention group (Figure 4A), and, in the control group, it changed from 14.3 ± 3.9 at T0 to 20.5 ± 5.1 at T1 and 14.1 ± 4.4 at T2. The between-group analysis showed a significant difference between the groups with respect to the change in RMS flexion at T1 (intervention group: ΔT1 = 13.3; control group: ΔT1 = 6.2; *p* = 0.016) and T2 (intervention group: ΔT2 = 11.5; control group: ΔT2 = −0.2; *p* = 0.004).

The RMS extension increased from 25.5 ± 7.1 at T0 to 36.2 ± 9.4 at T1 and 34.0 ± 8.0 at T2 in the intervention group (Figure 4B), while, in the control group, it changed from 26.2 ± 6.6 at T0 to 30.5 ± 8.9 at T1 and 25.3 ± 7.3 at T2. The between-group analysis showed a significant difference between the groups concerning the change in RMS extension at T1 (intervention group: ΔT1 = 8.5; control group: ΔT1 = 4.3; *p* = 0.040) and T2 (intervention group: ΔT2 = 8.5; control group: ΔT2 = −0.9; *p* = 0.004).

## 3. Discussion

Structured stretching exercises plus BONT/A injections effectively improved upper-limb post-stroke spasticity compared to self-stretching plus BONT/A injections over a 6-month period. The patients in the intervention group had a more significant reduction in post-stroke shoulder pain, were more independent in their daily living, had further improvements in their health-related quality of life, and showed a more significant reduction in RMS measured by EMG compared to those who performed self-stretching plus BONT/A injections (control group).

The structured stretching exercise program was initiated immediately after the BONT/A injections procedure. Stretching is not only beneficial in stroke rehabilitation but also essential for preventing injuries before starting any exercise. Stretching improves viscoelasticity at the muscle–tendon level and reduces motor neuron excitability [27]. In order to improve the extensibility of the joint and stretch tightened soft tissue again, stretching exercises need to be performed every day for at least three months [28]. In our study, when structured stretching exercises were performed immediately after BONT/A injection, the patients and their caregivers could clearly see the joints of the upper limbs, including those of the fingers, becoming soft. Since stretching exercises are not a rehabilitation therapy with a dose-dependent effect, excessive doses and time should be avoided to prevent injury [29]. Administering appropriate doses of structured stretching exercises in patients with upper-limb post-stroke spasticity for a relatively shorter time could increase patient compliance and enhance the treatment effect.

One of the major problems with upper-limb spasticity is that it causes severe pain in the muscles and joints of the upper extremities, especially in the shoulders [30]. Shoulder pain after a stroke is identified in about 30% of stroke survivors, and it is a predictor of poor outcomes, including motor outcomes, function, depression, and quality of life [31]. A shoulder stretching exercise program was included in this study, showing that structured stretching exercises plus BONT/A injections improved patients’ shoulder pain. However, BONT/A injection was not performed for the pectoralis major. A recent meta-analysis that included nine studies analyzed the effects of pectoralis major BONT/A injections on shoulder-pain relief and reported that relief in pectoralis major spasticity might contribute to reducing shoulder pain [32]. A randomized controlled study reported that BONT/A injections for the subscapularis and pectoralis muscles help increase the shoulder’s abduction and external rotation at rest during maximum passive and active movement [33]. However, BONT/A injections for shoulder girdle muscles were not considered in our study because the Korean government does not currently approve them. As a result, since upper-limb spasticity is one of the major causes of post-stroke shoulder pain, structured stretching exercises plus BONT/A injections can be assumed to alleviate shoulder pain by relieving spasticity. BONT/A injections for additional pectoralis major and subscapularis are expected to be more effective in relieving hemiplegic shoulder pain when combined with structured stretching exercises.

Both the patients who were assisted in their structured stretching exercises and received BONT/A injections and the patients who received instructions on self-stretching exercises plus BONT/A injections showed similar improvements in their motor function after 6 months. Although the International Consensus Statement declares that motor function improves in some patients after BoNT-A injections [34], the effect of BoNT-A on motor recovery has been controversial [35]. Many factors are involved in motor recovery in patients who have had a stroke, but many studies have suggested that the degree of motor function impairment in the early stages of a stroke is an important predictor [36]. The participants in this study had a mean FMA_UE score of 10. They had severe motor impairments after the stroke, so neurological impairment was thought to have had a more significant impact on their motor function recovery than the effect of spasticity relief. A systemic review extensively analyzed studies on the effectiveness of botulinum toxin therapy combined with rehabilitation, reporting that evidence of the effect of spasticity relief on motor recovery after a stroke is still insufficient [37]. 

Following these studies, two papers related to BoNT-A and upper-limb motor function recovery were published [35,38]. Hamaguchi et al. [38] examined the association between the recovery of upper-limb motor function and subjective symptoms such as muscle relaxation and pain after treatment in patients who continued BoNT-A treatment and upper-limb exercise therapy for more than five years after a stroke, suggesting that long-term BoNT-A treatment can improve the subjective symptoms of insomnia and motor weakness caused by stroke sequelae and reduce pain without the recovery of upper-limb motor function [38]. Hung et al. [35] performed a study to explore the predictors of clinically important changes in active motor function and the daily use of the affected upper limb following BoNT-A injection for upper-extremity spasticity after a stroke, reporting that patients less than 36 months post stroke were more likely to achieve a clinically significant improvement in upper-limb motor function. In addition, patients with a more extended post-injection period and greater proximal upper-extremity muscle strength could use the affected upper extremity more frequently in daily living activities [35]. Including these two additional recent studies is not yet enough to validate the hypothesis that BoNT-A may contribute to motor function recovery. Future studies are necessary to investigate the effects of structured stretching exercises and BONT/A injections on motor function recovery using additional assessment tools, including neuroimaging.

In this study, the participants were given BoNT/A injections in their muscles at a predetermined dose and then performed structured stretching or home-based self-stretching exercises. The Korean government has approved the use of up to 300 units of BONT/A to treat upper-limb spasticity after a stroke, a total dose which our patients received in their BB, BR, FDR, FCU, FDS, and FDP. The dosage and location of BONT/A used to treat upper-limb spasticity vary slightly depending on the researcher, but the BB, BR, FDR, FCU, FDS, and FDP are the most commonly chosen muscles for upper-limb spasticity treatment [39]. Based on the results of many studies, the manufacturer suggests the recommended dosage and injection muscles to compensate for the differences in the BONT/A injection site and dosage depending on the severity of spasticity in patients who have had a stroke, abnormal posture, and the doctor’s experience. The recommended dosage posted on the manufacturer’s website (https://www.botoxone.com/adult-spasticity/dosing, accessed on 26 May 2024) and the results of their Delphi panel were used to determine the target muscles and dosage in this study [40]. Due to the diversity in BONT/A injection techniques, when conducting additional intervention studies to enhance the effectiveness of BONT/A, there is a tendency to indicate only that the injection was given at the appropriate dose based on the patient’s condition [41,42]. Since the location and dose of BONT/A injections are factors which affect the effectiveness of the treatment, 300 units of BONT/A were injected into a predetermined area to reduce bias that could have affected the purpose of this study.

Several limitations exist in this study. An analysis of the treatment effect response by stroke type was not included. According to several studies [4], spasticity could be more severe in hemorrhagic strokes. However, studies examining the responsiveness of adjuvant therapies such as BONT/A or physical therapy depending on stroke type are still limited. However, the effect of spasticity relief from structured stretching exercises plus BONT/A injections might differ depending on the stroke type. In addition, the therapeutic effect of the afore-mentioned therapy based on brain lesion location was not investigated. Several studies have reportedly examined the association between the occurrence of spasticity after a stroke and brain lesions. For example, Lee et al. [43] have reported that brain lesions in the superior corona radiata, posterior limb of the internal capsule, posterior corona radiata, thalamus, putamen, premotor cortex, and insula are associated with the development of upper-limb spasticity using the voxel-based lesion symptom-mapping method. However, research into the differences in the brain lesion-specific responsiveness to BONT/A injections for spasticity is still limited. Cost-effectiveness analyses of structured stretching exercises plus BONT/A injections for patients who have had a stroke are limited due to the cost differences between individual countries due to their health policies and healthcare providers. Therefore, these limitations should be addressed to obtain highly reliable results in future studies.

## 4. Conclusions

Structured stretching exercises plus BONT/A injections for six months showed a superior effect in relieving post-stroke upper-limb spasticity compared to self-stretching exercises plus BONT/A injections. These exercises, which began immediately after the BONT/A injections, contributed to patients’ and caregivers’ increased adherence to the program. The proposed rehabilitation protocols following BONT/A injections effectively improved post-stroke shoulder pain, independence in functional ADL, and health-related quality of life in the long term. The structured stretching exercise plus BONT/A injection protocol proposed in this study is expected to be helpful in clinical practice for patients who have had a stroke with upper-limb spasticity scheduled to receive BONT/A injections.

## 5. Materials and Methods

### 5.1. Study Design

This study was designed as a prospective, randomized, double-blinded, controlled trial with participants randomly assigned to the intervention and control groups at a 1:1 ratio. Randomization was performed using computer-generated, randomly permuted blocks in a pseudorandom sequence. Measurement collection at the hospital and data analysis were both performed by researchers blinded to the group allocations. However, blinding the participants or therapists to the group allocations was not possible.

We recruited patients who had experienced a stroke an upper-limb muscle (the elbow, wrist, or fingers) spasticity of grade 2 or higher on the Modified Ashworth Scale (MAS) who were scheduled to receive BONT/A injections. This intervention was conducted in patients aged 20–79 years who had suffered subacute strokes in the previous 6 months. Subjects in whom botulinum toxin injection was contraindicated due to neuromuscular disorders, toxin allergy, pregnancy, and infection were excluded [44]. In addition, patients who had already experienced a prior BONT/A injection, upper-limb injuries, and severe cognitive impairments were excluded.

The patients’ demographic information, including age, sex, stroke type, lesion location, lesion side, duration between stroke onset and treatment, comorbidities, National Institutes of Health Stroke Scale (NIHSS) score at the time of emergency room admission for stroke, score on the Korean version of the Mini-Mental State Exam (K-MMSE), and MAS score of the most severely spastic upper-limb muscles, was collected before intervention initiation.

### 5.2. BONT/A Injection Methods

BONT/A (BOTOX^®^; AbbVie Inc., North Chicago, IL, USA) was standardized by diluting one vial (100 units/vial) with 2.0 mL of a 0.9% sodium chloride solution (5.0 U/0.1 mL). Injections were performed intramuscularly, with a total of 300 U of BONT/A at predetermined doses in all six muscles of the affected upper limb using electromyography (EMG) guidance (NICOLET EDX, Natus Neurology Inc., Middleton, WI, USA).

The South Korean government has approved a maximum dose of 300 UI BONT/A that can be used to treat post-stroke upper limb spasticity every six months. When doctors decide the dose and the sites of BONT/A to treat upper limb spasticity in stroke patients, they must consider the patient’s condition and the country-specific BONT/A authorizations. It is necessary to consider various situations, such as abnormal posture caused by spasticity of the muscles of the upper limb of stroke patients, the severity, and the size of the muscles. However, BONT/A injections were given at predetermined sites and doses since this study focused on structured stretching. The authors determined the dosages and locations of the BONT/A injections, shown in Table 2, based on the methods and Delphi panel recommendations presented by AbbVie (https://www.botoxone.com/adult-spasticity/dosing accessed on 26 May 2024 and [40], respectively).

EMG ensured needle placement accuracy by recording muscle activity during active or passive movements or observing the movements during electrical stimulation of the muscle. BONT/A was injected into the patients using a special electrode needle (Myoject™: Natus Neurology Inc., Middleton, WI, USA), which allowed for the drug to be injected while simultaneously checking the EMG signals.

### 5.3. Structured Stretching Exercises Plus BONT/A Injections

**Figure 5:**
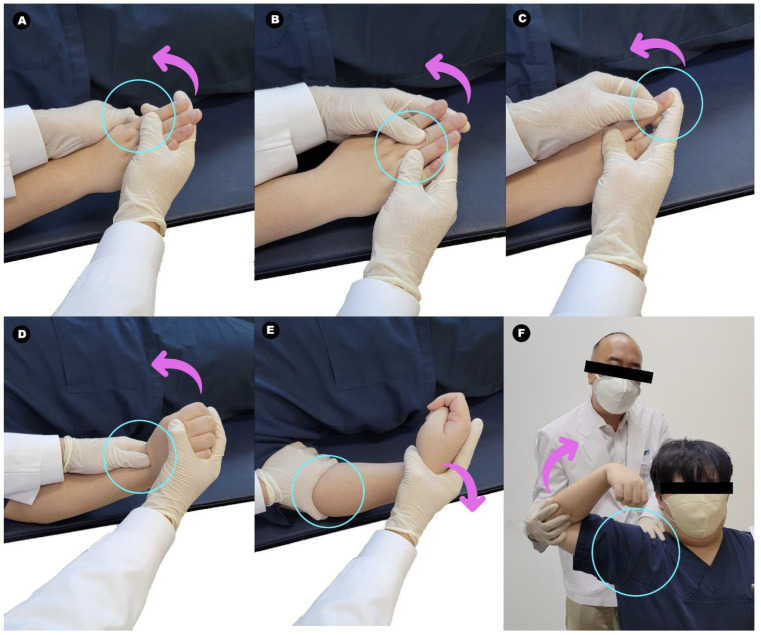
Structured stretching exercises after botulinum toxin A (BONT/A) injection. (Blue circle: Target joints. Pink arrow: Directions for stretching.) The patient is assisted in performing stretching exercises immediately after their BONT/A injections while lying down. Starting with the finger joints, each joint is slowly stretched at a low intensity in the opposite direction of the bend. When the patient reaches the maximum angle at which the pain is tolerable, the position is held for 2 s, and then the muscle is relaxed. Each upper-limb muscle is stretched individually to avoid pain. The physiatrist holds the patient’s scapular in place. Then, by slowly rotating the shoulder, the shoulder girdle muscles are stretched clockwise or counterclockwise to an angle that the patient can tolerate. (**A**) Metacarpophalangeal joint; (**B**) proximal interphalangeal joint; (**C**) distal interphalangeal joint; (**D**) wrist joint; (**E**) elbow joint; and (**F**) shoulder joint.

Patients of the intervention group were assisted in performing structured stretching exercises immediately after their BONT/A injections, while lying down. Since all the muscles of the patients’ wrists, fingers, and elbows were tightly bent due to spasticity, the joints of their wrists and fingers had to be stretched slowly and at a low intensity in the opposite direction of the bend (Figure 5).

When the maximum angle at which the patient could tolerate pain was reached, the position was held for 2 s, and then the muscle was relaxed [25]. To avoid pain from stretching a single joint for a long time, the wrist, metacarpophalangeal (MCP), proximal interphalangeal (PIP), and distal interphalangeal (DIP) joints were stretched individually. Then, elbow stretching was performed by extending the elbow joint to its maximum range of motion, holding it for 2 s, and then releasing it. The therapist kept the elbow joint in a fixed position to prevent pronation and supination of the upper arm and focused on flexion and relaxation of the patient’s elbow joint. Thereafter, the patient sat in a wheelchair or chair, and shoulder-muscle stretching was performed [29]. To prevent the scapular from rotating, the therapist held it in place. Then, by slowly rotating the shoulder, the shoulder girdle muscles were stretched clockwise or counterclockwise to an angle that the patient could tolerate.

The patients in the intervention group were given assistance with these structured stretching exercises for 20 min per day by a physiotherapist blinded to the group assignments. To improve compliance, the patients and caregivers were given directions to feel the muscles around the finger, wrist, elbow, and shoulder joints after the upper-limb stretching exercises to confirm the changes in muscle quality. Meanwhile, the control patients were provided a brochure with instructions on upper-limb self-stretching exercises to conduct at home. They were advised to perform these for the same amount of time as the intervention group (20 min per day) to guarantee the same treatment dose and were provided with a checklist to routinely self-monitor compliance, either themselves or via their caregivers.

The intervention and control patients performed, respectively, structured stretching exercises and self-stretching exercises five times a week for six months after the BONT/A injections. Because excessive rehabilitation therapy, including stretching exercises, can cause injury and pain to patients, all the participants were asked not to perform additional stretching exercises beyond the duration and methods prescribed in this study.

Conventional stroke rehabilitation therapies, such as physical and occupational therapy, were also administered by therapists not involved in this study and maintained for 1 h per day in outpatient settings for both groups of patients during the study period but did not include upper-limb stretching.

### 5.4. Outcome Measures

The degree of spasticity in the upper-limb joint muscles, including fingers, elbows, and shoulders, was measured via the MAS (modified and assessed on a scale of 0–5 to facilitate treatment effect analysis, see Table 3) and used as the primary outcome when comparing the effect of stretching exercises after BONT/A injection on spasticity relief between the groups.

The secondary outcome indicators were the level of pain in the affected shoulder, ADL, upper-limb motor function, and health-related quality of life. The Visual Analogue Scale (VAS) scores were measured to investigate the stretching exercises’ effect after BONT/A injection on relieving post-stroke shoulder pain [31]. The VAS comprises 10 levels, from 1 (no pain) to 10 (worst pain), as shown from left to right. Korean versions of the Modified Barthel Index (K-MBI) and Fugl-Meyer Assessment of Upper Extremities (FMA_UE) were used to determine whether this intervention contributed to improvements in the ADL and motor recovery of the upper limbs.

K-MBI is an evaluation tool for assessing the independence of daily living activities. K-MBI consists of 10 evaluation items (personal hygiene, bathing, eating, toileting, stair climbing, dressing, defecation, voiding, walking, and chair–bed transfer), the scores of which are individually divided into five phases by item, and nine weights are applied depending on content proportion [45]. The total score is 100 points: the higher the score, the more independent the patient can be in their daily lives. This method’s inter-rater reliabilities range from 0.93 to 0.98, and Cronbach’s alpha, as K-MBI’s internal consistency reliability, is 0.84 [46]. The FMA_UE is a widely used tool to evaluate sensorimotor impairment in patients who have had a stroke, whereby each item is evaluated on a three-point scale, and the maximum possible score is 66 points [45,47].

The health-related quality of life was assessed using the EuroQol-5 Dimension (EQ-5D), a questionnaire which provides a patient’s self-reported health status, comprising five items: mobility, self-care, usual activity, pain and/or discomfort, and anxiety and/or depression [48]. EQ-5D index scores range from −0.59 to 1, where 1 is the best possible health state. EQ-5D results are displayed in 3 decimal places (e.g., 0.912), making it readers challenging to understand. The original score is multiplied by 100 and displayed as a graph to make it easier for readers to grasp the results.

In addition, EMG was used to quantitatively analyze this intervention’s effect on post-stroke spasticity, processing the EMG signals using the MATLAB R2021b software (Math Works Inc., Natick, MA, USA). Indeed, a method of analyzing the level of spasticity by detecting the electromyographic signals generated through surface electrodes attached to the biceps and triceps when passively extending and flexing a patient’s elbow joint was recently proposed [49]. In brief, using the root mean square (RMS) values measured with EMG in the biceps and triceps, the RMS index at flexion and extension was calculated using the following formula: RMS index = Agonist RMS/(Agonist RMS + Antagonist RMS) [49]. This method has been reported to significantly correlate with the level of spasticity measured using the MAS in patients who have had a stroke [49].

All the primary and secondary behavioral assessment and electromyography indicators were assessed three times: before (T0) and three months (T1) and six months after (T2) the intervention.

### 5.5. Statistics

To calculate the sample size needed to compare the primary outcome measures between the groups in G*Power 3.1.9.7, we used a two-tailed test with an effect size (d) of 1.0, an α error of 0.05, and a power of 0.8, indicating the need for 17 participants in each group. Considering a 20% chance of dropout due to outpatient visits, at least 22 patients from each group were required to participate in this study.

The Kolmogorov–Smirnov test was used to verify the normal distribution of data. An independent *t*-test or a Mann–Whitney U test for the continuous variables and a chi-square test or Fisher’s exact test for the categorical variables were used to determine differences in the baseline parameters between the two groups. A repeated measures analysis of variance (RM-ANOVA) was used to evaluate the time and group interaction effects to indicate significant differences in the degree of change over time between the groups. A post hoc analysis was performed with a Tukey correction. Changes in each variable from before to after the intervention were analyzed within the groups using a paired *t*-test. An independent *t*-test was performed to compare the therapeutic effects between the groups. A *p*-value below 0.05 was defined as statistically significant, and all the statistical analyses were performed using SPSS Statistics v.29.0 (IBM SPSS Statistics for Windows, IBM Corp., Armonk, NY, USA).

## Figures and Tables

**Table 1:** Demographic and clinical characteristics of the participants.

		Intervention Group (*n* = 21)	Control Group (*n* = 22)
Age	(years, mean ± SD)	66.3 ± 8.2	68.1 ± 8.4
Sex	Male	9	10
	Female	12	12
Stroke type	Infarct	14	15
	Hemorrhage	7	7
Hemiplegic side	Right	8	9
	Left	13	13
Duration after stroke onset	(days)	71.1 ± 9.1	74.8 ± 9.2
Comorbidity	Hypertension	20	20
	Diabetes	11	12
	Hyperlipidemia	16	17
NIHSS	Onset	14.1 ± 3.0	13.9 ± 3.1
K-MMSE	(at the beginning of the study)	19.3 ± 4.0	18.5 ± 4.5
Modified MAS	0	0	0
grade	1	0	0
	2	0	0
	3	14	16
	4	7	6
	5	0	0

Values are presented as a number (%) or mean ± standard deviation. NIHSS, National Institutes of Health Stroke Scale; MAS, Modified Ashworth Scale; and K-MMSE, Korean version of the Mini-Mental State Exam.

**Table 2:** Sites and doses of botulinum toxin A injection.

Muscles (BONT/A, UI)	This Study	AbbVie Website	Allergan Delphi Panel
Biceps brachii (BB)	90	60–200	0–50
Brachioradialis (BR)	45	45–75	25–50
Flexor carpi radialis (FCR)	30	12.5–50	50–75
Flexor carpi ulnaris (FCU)	30	12.5–50	25–50
Flexor digitorum superficialis (FDS)	45	30–50	20–60
Flexor digitorum profundus (FDP)	60	30–50	25–75

BONT/A, Botulinum toxin A.

**Table 3:** The modified scoring system of the Modified Ashworth Scale.

Modified Score	Actual Score	Modified Ashworth Scale
0	0	No increase in muscle tone
1	1	Slight increase in muscle tone, manifested by a catch and release or by minimal resistance at the end of the range of motion (ROM) when the affected part is moved in flexion or extension
2	1+	Slight increase in muscle tone, manifested by a catch, followed by minimal resistance throughout the remainder (less than half) of the ROM
3	2	More marked increase in muscle tone through most of the ROM, but affected parts easily moved
4	3	Considerable increase in muscle tone, passive movement difficult
5	4	Affected part rigid in flexion or extension

## Data Availability

The datasets analyzed during the current study are available from the corresponding author on reasonable request. The data are not publicly available due to privacy or ethical restrictions.
